# The epidemiology of multiple sclerosis in the entre Douro e Vouga region of northern Portugal: a multisource population-based study

**DOI:** 10.1186/s12883-020-01755-8

**Published:** 2020-05-19

**Authors:** Mariana Branco, Ivânia Alves, Ana Martins da Silva, Joaquim Pinheiro, Maria José Sá, Inês Correia, Lívia Sousa, Eva Brandão, Carlos Veira, Bernardo Gomes, Luis Ruano

**Affiliations:** 1 Serviço de Neurologia, Centro Hospitalar de Entre Douro e Vouga, Santa Maria da Feira, Portugal; 2 Departamento de Ciências da Saúde Pública e Forenses e Educação Médica, Faculdade de Medicina, Universidade do Porto, Porto, Portugal; 3 Serviço de Neurologia, Centro Hospitalar do Tâmega e Sousa, Penafiel, Portugal; 4 Serviço de Neurologia Hospital de Santo António, Centro Hospitalar Universitário do Porto, Porto, Portugal; 5 Unidade Multidisciplinar de Investigação Biomédica (UMIB), Instituto Ciências Biomédicas Abel Salazar, Universidade do Porto, Porto, Portugal; 6 Serviço de Neurologia, Centro Hospitalar de Vila Nova de Gaia/Espinho, Vila Nova de Gaia, Portugal; 7 Serviço de Neurologia, Centro Hospitalar de São João, Porto, Portugal; 8 Faculdade de Ciências da Saúde, Universidade Fernando Pessoa, Porto, Portugal; 9 Serviço de Neurologia, Centro Hospitalar e Universitário de Coimbra, Coimbra, Portugal; 10 Unidade de Saúde Pública, ACES Entre Douro e Vouga I, Santa Maria da Feira, Portugal; 11 EPIUnit - Instituto de Saúde Pública, Universidade do Porto, Porto, Portugal

**Keywords:** Multiple sclerosis, Epidemiology, Prevalence, Portugal

## Abstract

**Abstract:**

**Background:**

The prevalence of Multiple Sclerosis (MS) has been increasing worldwide and the north–south gradient of prevalence may be disappearing in the Northern hemisphere. The few previous prevalence studies performed in Portugal have reported a lower prevalence than the average for Western Europe. The aim of this study is to estimate the prevalence of MS in the Entre Douro e Vouga region, in Northern Portugal.

**Methods:**

Multiple overlapping sources were used to ascertain all cases from the reference population: records from hospitals in the region and neighbouring regions; diagnostic databases of primary care physicians; and applications for disability benefits. The prevalence date was set at 1 January 2014. The reference population was 274,859 inhabitants. Patients’ neurologists were contacted to retrieve clinical information and confirm the diagnosis based.

**Results:**

A total of 177 patients were identified after eliminating duplicates from different sources. The female to male ratio was 1.9 and the mean age at disease onset was 33.5 (standard deviation: 10.3). Clinically isolated syndrome accounted for 9.0% of patients, relapsing remitting for 58.8%, secondary progressive for 20.3% and primary progressive for 11.8%. The prevalence was estimated in 64.4 patients per 100,000 (95% confidence interval: 54.9;73.9).

**Conclusions:**

In this study we report a higher point prevalence of MS than had been previously described in Portugal, but still far from the higher values recently reported in other Southern European countries.

## Background

Multiple sclerosis (MS) is a chronic inflammatory disease of the central nervous system. The early onset and the progressive disabling course of the disease cause a major impact on patients’ life and represent a relevant social and economic burden in western societies [1, 2].

The uneven distribution of MS across the globe has puzzled epidemiologists and neurologists for almost a century. A latitudinal gradient of the prevalence of MS has long been described [3, 4]. However, a systematic review of prevalence studies suggested that this prevalence gradient could be disappearing in Northern America and Western Europe, while it persists in Australia and New Zealand [5]. Although these findings were not confirmed in a second systematic review [6], more recent studies from Southern Europe have reported prevalence values similar to the ones usually described in Northern European countries [7–10].

Epidemiological data regarding MS in Portugal are scarce. A population-based study performed in 1998 in the region of Santarem, in central Portugal, reported a prevalence of 46.3 per 100,000 [11]. Another population based performed in 2009 identified a crude prevalence of 41.4 per 100,000 in three primary-care districts from Lisbon [12]. A third prevalence study, also performed in 2009, yielded a prevalence of 39.8 per 100,000 in the district of Braga, but relied only in hospital records for case identification [13]. Currently, there is no published population-based epidemiological data on the female to male ratio, age of onset, and the distribution of the different clinical courses of MS in Portugal.

This study aims to describe the epidemiology and prevalence of MS in the region of Entre Douro-e-Vouga in Northern Portugal, using a multisource population-based approach.

## Methods

### Study design and setting

This is a multi-source population-based study on the prevalence and epidemiology of MS in Entre Douro e Vouga region. The study was approved by the ethics committee of the Regional Health Authority for Northern Portugal, that also gave permission to waive individual informed consent for data access, with all the patient data de-identified prior to data collection.

The Entre Douro e Vouga region is a well-defined geographical region located in Northern Portugal, classified as a Level III region in the European Nomenclature of Territorial Units for Statistics. The Entre Douro e Vouga region spans over urban, suburban and rural areas and has an area of 861 km^2^ and a population of 274.859, including the counties of Arouca, São João da Madeira, Santa Maria da Feira, Vale de Cambra, and Oliveira de Azemeis. Within this region, there are 26 primary care units, where 159 General Practitioners (GPs) work. The coverage and usage of the Portuguese National Health Service (NHS) is extensive in the Portuguese population. GPs refer patients with neurological disorders to a single reference hospital for neurological disorders within the NHS. However, this hospital was created 20 years ago, and previously diagnosed patients with MS could be followed in hospitals from larger cities in the nearby regions. There are no private hospitals with neurology departments in the region. The immunomodulatory drugs used in the treatment of MS are restricted to hospital use in Portugal and are fully refundable through the NHS; therefore, they are hardly ever used outside NHS hospitals.

### Patient ascertainment methods

Patient ascertainment was performed from three potentially overlapping sources: 1) Hospital records, 2) Primary care diagnostic databases and 3) Applications for disability benefits. In the next paragraphs, we detail how patient search was performed in each of the sources.

1. Hospital records: Patient search was performed in the only referring hospital for neurological disorders in the region and in hospitals from nearby regions with a Neurology department. In each hospital, the records of the neurology outpatient clinics were reviewed to identify all patients with MS living in the Entre Douro e Vouga region.
2. Primary care diagnostic databases: The coding system used to classify patients in primary care settings in Portugal is the electronic version of the International Classification of Primary Care, second edition [14]. The electronic databases of all primary care units within the Entre Douro e Vouga region were searched for the code N86 (multiple sclerosis).
3. Applications for disability benefits: To receive tax benefits or exemption of co-payments patients with MS must apply for a medical evaluation in the Public Health Unit of the area of residence. The application records for medical evaluations in the Entre Douro e Vouga public health department from October 2009 to July 2014 were reviewed to identify applicants with possible MS.

The period of patient ascertainment was from January to July 2014. To identify and discard duplicates, the NHS number, the current address and date of birth were retrieved for each patient, using an algorithm to anonymize and match identical records.

### Inclusion criteria

For all the unique records identified through the different sources, the patient’s neurologist was contacted and asked to provide clinical information and confirm a diagnosis of MS on the 1st of January 2014, based on the 2017 McDonald criteria [15]. The patients were confirmed to be alive at the prevalence date trough analysis of recent visits to primary and hospital care and death certificates.

### Clinical data collection

Patients’ neurologists were asked to review the patients clinical records and provide additional data: sex, age of onset, disease duration, physical disability according to the Expanded Disability Status Scale (EDSS) [16] and current clinical form of the disease: 1) clinically isolated syndrome (CIS) 2) relapsing remitting (RR) 3) secondary progressive (SP) or 4) primary progressive (PP) according to the 2013 revision of the Lublin criteria [17].

### Data analysis

The mean age, age of onset and median EDSS were calculated for the whole sample and each disease course. Prevalence is reported as the number of patients per 100,000. It was age-standardized using the direct method. Data from the last census, in 2011 were used to estimate the population of the Entre Douro e Vouga region and to define the Portuguese reference population [18, 19]. For the European population, the European Standard Population 2013 [20] was used. An estimate of the overall prevalence, corrected using the capture-recapture method was also calculated, using the maximum likelihood estimator methodology [21]. Retrospectively estimated incidence rates were calculated for the three-year periods of 2005–2007, 2008–2010 and 2011–2013. The statistical analysis was performed using SPSS 24.0.

## Results

**Fig. 1:**
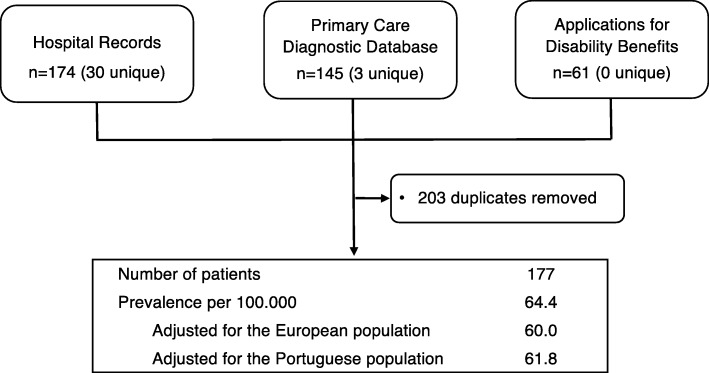
Flow chart and prevalence data

A total of 174 patients where identified from hospital records. All the 5 public hospitals that have Neurology departments located in the nearby regions collaborated in the study. Patients living in the Entre Douro e Vouga region were identified in 4 of these 5 hospitals. A total of 172 records were retrieved from the primary care diagnostic databases, 145 of them confirmed to have MS, the others corresponding to coding errors. Regarding applications for disability benefits, 61 patients were identified, all with confirmed MS by their Neurologist and previously identified by hospital records (Fig. 1).

A total of 177 unique patients were identified after eliminating duplicates, yielding a crude prevalence estimate of 64.4 patients per 100,000 with a 95% confidence interval (CI) of 54.9 to 73.9 per 100,000. The age-standardized prevalence for the Portuguese population was 61.8 per 100,000 and for the European standard population 60.0 per 100,000.

The retrospectively estimated incidence rate from 2005 to 2007 was 2.5 (95% CI: 1.6; 3.9), from 2008 to 2010 it was 2.7 (95% CI: 1.7:4.0) and from 2011 to 2013 it was 3.1 new patients per 100,000 person-years (95% CI: 2.1;4.6).

For the capture-recapture analysis, we used the two sources that provided unique cases 1) hospital records and 2) primary care diagnostic databases, as the third source (applications for disability benefits) was not suitable for this method, as it did not yield cases not independently identified from hospital records (Fig. 1). The probability of case identification by hospital records was 0.98; the probability of identification from primary care diagnostic databases was 0.80. The expected number of overlapping cases was 139.1, similar to the observed number (142). The number of total estimated patients was 181.4 and the prevalence adjusted by this method was 66.0 patients per 100,000.

The patients’ mean age was 44.5 (standard deviation (SD): 11.9) and the median EDSS was 2.0 (interquartile range: 1.0; 6.0). CIS course accounted for 9.0% of patients, RR course for 58.8% of patients, SP for 20.3% and PP for 11.9%. The mean age at onset was 33.5 (SD: 10.3), 32.4 in patients with CIS, 31.4 in RR, 35.0 in SP and 42.9 in PP. The female to male ratio was 1.9 overall, 2.0 in patients with CIS, 1.9 in RR, 1.4 in SP and 1.2 in PP (Table 1).

**Table 1:** Clinical and demographic characteristics of the study patients

	CIS (*n* = 16)	RR (*n* = 104)	SP (*n* = 36)	PP (*n* = 21)	Total sample (*n* = 177)
Age, mean (SD), *years*	35.5 (7.6)	41.9 (11.7)	54.2 (10.3)	54.2 (10.2)	44.5 (11.9)
Sex ratio (female/male)	2.0	1.9	1.4	1.2	1.9
Age at onset, mean (SD), *years*	32.4 (6.0)	31.4 (10.0)	35.0 (10.7)	42.9 (9.2)	33.5 (10.3)
Disease duration, mean (SD), *years*	4.5 (3.9)	10.8 (6.8)	16.7 (9.5)	11.7 (7.1)	11.5 (7.9)
EDSS, median (IQR)	1.0 [0.0; 2.0]	2.0 [1.0; 3.0]	5.0 [4.0; 6.5]	6.5 [5.5; 7.5]	2.0 [1.0; 6.0]

*CIS* clinically isolated syndrome; *RR* relapsing remitting; *SP* secondary progressive; *PP* primary progressive; *SD* standard deviation; *IQR* interquartile range *EDSS* expanded disability status scale

## Discussion

In this multi-source population-based study we estimated a prevalence of MS of 64.4 patients per 100,000, higher than previously described in Portugal (Table 2), but still far from the highest values recently reported in other southern European countries [22].

**Table 2:** Studies on the prevalence of multiple sclerosis in Portugal

Author	Year	Design	Pop.	Area	N	Prevalence per 100,000 [95% CI]
De Sá, J [11].	1999	Multisource population-based	62,621	Santarem District	29	46.3 [29.5–63.2]
De Sá, J [12].	2009	Multisource population-based	229,342	Lisbon (three parishes)	95	41.4 [33.5–50.6]
Figueiredo J [13].	2009	Hospital based	866,012	Braga district	345	39.8 [27.5–52.2]
Branco, M (present study)	2014	Multisource population-based	274,859	Entre Douro e Vouga Region	177	64.4 [54.9–73.9]

The previous prevalence studies in Portugal, performed a decade or more ago, reported figures ranging from 39.8 to 46.3 patients per 100,000 [11–13]. However, we must be cautious when comparing these results. The inclusion of patients in some of the previous studies relied on older diagnostic criteria, excluding patients with CIS. Nevertheless, even when removing these patients from our current estimate, the point prevalence is still higher, at 58.6 patients per 100,000 (CI: 50.0; 68.0). However, the confidence interval overlaps with the Santarem study, with 46.3 patients per 100,000 (CI: 29.5–63.2). Additionally, the higher prevalence in this study could reflect a better case finding when compared with previous studies, due to the use of multiple sources and records from hospitals in nearby regions.

While the study was not designed with this goal, we performed a *post-hoc* analysis of the data to estimate incidence trends. There was a trend to an increase in incidence, but with no statistical significance. The estimated incidence is lower than generally described in Europe, in line with the prevalence figs [23]..

The multisource design of this study and the extensive cooperation of MS centres from contiguous regions gives us confidence that we were able to identify most of the affected patients in the region. Nevertheless, older and more disabled patients, particularly those with progressive courses of disease, are less prone to have a regular follow up at hospital clinics when compared with younger, less disabled patients with CIS or RR course. While we tried to identify those patients using primary health care databases and applications for disability benefits, there were probably missed cases resulting in an overall underestimation of the prevalence of the disease. The relatively low median EDSS of the overall sample (2.0), when compared to what is generally reported for prevalent cases [24–26], is possibly a reflection of a lower ascertainment rate of older and more disabled patients in the present studies. Nevertheless, we identified a larger number of patients with primary progressive course (SP 20.3%; PP 11.9%) when compared with previous Portuguese studies (Santarem: SP 20.1% and PP 3.4%; Braga: SP 14.2% and PP 2.0%). Concerning the Braga study, the lower prevalence of progressive course is in agreement to a lower mean age in that sample, 35.4 vs. 44.5 in the present study. The Santarem study sample had a similar mean age, but its small dimension (29 patients) limits the comparisons. Early preliminary data from an ongoing project to build a national MS registry from an initial pool of 561 patients from several MS centres identified 8.6% of patients as PP and 10.4% as SP [27].

We performed a capture-recapture analysis of the prevalence data using previously described methodology [21]. However, we must interpret this result caution, given as the assumption of source independence Indeed, it is expected that patients that have a regular follow-up at the MS clinic (first source) are more likely to attend and have a correct diagnosis registered at a Primary Care (second source). Indeed, only two sources that provided unique cases 1) hospital records and 2) primary care diagnostic databases, as the third source (applications for disability benefits) did not yield any cases that were not independently identified from hospital records. This is similar to the unadjusted prevalence, due to the completeness of the hospital sources, that identified 174 of the 177 unique patients.

On the assumption that the prevalence in the Entre Douro e Vouga region is higher than in other regions of Portugal, we can speculate on some hypotheses. Recent research strongly suggests that organic solvents could play a role in the development of MS [28], and these compounds are widely used in footwear manufacturing [29]. This region has an important footwear industry [30] and shoe factories are remarkable local employers, which makes us hypothesize that there is a higher number of people exposed to organic solvents than in other regions of the country and this may eventually contribute to a higher prevalence of MS. Additional studies considering the professional activities of patients are needed to clarify this issue.

While recent studies in southern Europe have reported prevalence values similar to the ones usually described in northern European countries [7–10], the prevalence reported in this study, and indeed in previous Portuguese studies, is still lower than the European average [22]. Some factors may help to explain this lower prevalence. Vitamin D is a well-known protective factor against the development of MS [31]. Portugal is a small country with an extensive coastline, and the large majority of the population is concentrated in the cost, having easy access to bathing areas. In larger countries like Spain and Italy there is a higher proportion of the population that lives far from the coast and, and probably a lower average number of annual hours of sunbathing. The Portuguese population has a higher prevalence of sunburns compared to other southern countries, namely Italy and Greece, supporting the hypothesis of a more intense sun exposure [32]. Nevertheless, there is still an important vitamin D deficiency in the Portuguese population [33], and particularly in patients with MS [34]. Ethnic and racial distribution could also play a role in these results. African populations have a low prevalence of MS [35] and Portugal is historically one of the main destination countries for African migrants in Southern Europe [36].

The female to male ratio was 1.9, lower than what has been reported in Northern European countries [5], where this ratio has been increasing, reflecting an increasingly higher relative incidence in females [37, 38]. One potential explanation for this increase is the loss of the relatively long-lasting protective factor of pregnancy since there is a trend to lower birth rates. However, this trend is even more pronounced in Portugal than in Northern European countries [39]. There are currently no other factors able to justify the gender differences in the epidemiology of MS [40], therefore we can only suppose that genetic or environmental factors might play a role on this difference, with Portuguese women having a more protective profile than their northern counterparts, or Portuguese men being more susceptible.

## Conclusions

In conclusion, we report the highest point prevalence of MS in a study performed in Portugal. This could be due to regional variance, differences in study methodology and inclusion criteria, a higher number of diagnosis due to an increased awareness of physicians about the disease, or environmental and genetic factors. Further studies are needed to truly understand the trends in the epidemiology of MS in Portugal.

## Abbreviations

MS: Multiple sclerosis
CI: Confidence interval
GPs: General Practitioners
NHS: Portuguese National Health Service
EDSS: Expanded Disability Status Scale
CIS: Clinically isolated syndrome
RR: Relapsing remitting
SP: Secondary progressive
PP: Primary progressive
